# Reduced Number of Adipose Lineage and Endothelial Cells in Epididymal fat in Response to Omega-3 PUFA in Mice Fed High-Fat Diet

**DOI:** 10.3390/md16120515

**Published:** 2018-12-18

**Authors:** Katerina Adamcova, Olga Horakova, Kristina Bardova, Petra Janovska, Marie Brezinova, Ondrej Kuda, Martin Rossmeisl, Jan Kopecky

**Affiliations:** Department of Adipose Tissue Biology, Institute of Physiology of the Czech Academy of Sciences, Videnska 1083, 142 20 Prague, Czech Republic; katerina.adamcova@fgu.cas.cz (K.A.); olga.horakova@fgu.cas.cz (O.H.); kristina.bardova@fgu.cas.cz (K.B.); petra.janovska@fgu.cas.cz (P.J.); marie.brezinova@fgu.cas.cz (M.B.); ondrej.kuda@fgu.cas.cz (O.K.); martin.rossmeisl@fgu.cas.cz (M.R.)

**Keywords:** cellularity, adipocyte, obesity, nutrition, fat, proliferation, white adipose tissue

## Abstract

We found previously that white adipose tissue (WAT) hyperplasia in obese mice was limited by dietary omega-3 polyunsaturated fatty acids (omega-3 PUFA). Here we aimed to characterize the underlying mechanism. C57BL/6N mice were fed a high-fat diet supplemented or not with omega-3 PUFA for one week or eight weeks; mice fed a standard chow diet were also used. In epididymal WAT (eWAT), DNA content was quantified, immunohistochemical analysis was used to reveal the size of adipocytes and macrophage content, and lipidomic analysis and a gene expression screen were performed to assess inflammatory status. The stromal-vascular fraction of eWAT, which contained most of the eWAT cells, except for adipocytes, was characterized using flow cytometry. Omega-3 PUFA supplementation limited the high-fat diet-induced increase in eWAT weight, cell number (DNA content), inflammation, and adipocyte growth. eWAT hyperplasia was compromised due to the limited increase in the number of preadipocytes and a decrease in the number of endothelial cells. The number of leukocytes and macrophages was unaffected, but a shift in macrophage polarization towards a less inflammatory phenotype was observed. Our results document that the counteraction of eWAT hyperplasia by omega-3 PUFA in dietary-obese mice reflects an effect on the number of adipose lineage and endothelial cells.

## 1. Introduction

An unhealthy lifestyle, including overnutrition, is the main driving force behind the recent pandemic of obesity and associated diseases. Obesity is defined as an excessive accumulation of body fat, namely in the form of white adipose tissue (WAT; [1]). This tissue is characterized by extreme plasticity, and fat depot-specific functional and structural heterogeneity (reviewed in [2,3,4]). The main function of WAT is to store energy in triglycerides that are located within lipid droplets in adipocytes. During exercise, fasting, or cold exposure, fatty acids are released and serve as an energy source. Sufficient capacity for WAT expansion is essential to prevent a spillover of fatty acids and lipotoxic damage of insulin signalling in other tissues [5]. Therefore, both an insufficient amount of WAT as well as hypertrophic WAT can lead to harmful systemic metabolic consequences. 

The growth of WAT can occur by both increasing the number of adipocytes (“hyperplasia“) and by enlarging the size of existing adipocytes (“hypertrophy“). WAT can represent 5% to 60% of total body weight [4,6]. Fat mass reflects the energy balance. However, the adipocyte number is very static in adult humans and independent of fluctuations in body weight, even in response to a massive weight loss. Furthermore, adipocyte number is set during childhood and adolescence [6] and approximately only 10% of fat cells are renewed annually in adult humans [7]. New adipocytes arise from adipogenic progenitor cells, as mature adipocytes are postmitotic [8]. Adipocyte progenitors are CD24^+^ cells, which lose their CD24 expression as they become further committed to the adipocyte lineage. The CD24^−^ preadipocytes represent the next distinct cell type over the course of adipose cell differentiation into mature adipocytes in vivo [9]. Adipose tissue expansion also involves coordinated development of the tissue vascular network and coupled angiogenesis is essential for adipogenesis in obesity [10,11]. In adult humans, obesity is predominantly associated with the hypertrophy of fat cells. However, an increase in fat cell number was also observed in morbidly obese subjects (reviewed in [6,7,12]). In contrast, in laboratory rodents WAT hyperplasia could be induced independent of age, e.g., in response to obesogenic high-fat diets. In particular, the epididymal WAT (eWAT) in the abdomen, the typical WAT depot in rodents (regarding its growth in response to high fat diet [13], negligible capacity for *Ucp1* expression [14] and its frequent analysis in the rodent studies focused on obesity [15]), has a high potential for hyperplastic growth [13,16,17,18].

WAT is composed of several cell types including adipocytes, preadipocytes (see above), and endothelial cells as well as fibroblasts, stem cells, and almost the full spectrum of immune cells defining a unique adipose-resident immune system [19]. Macrophages accumulate in the hypertrophied WAT of both obese individuals and mice, and divert from the pro-resolving (M2) to the pro-inflammatory phenotype (M1), which contribute to a low-grade adipose tissue inflammation and insulin resistance in obesity [20]. Mutual interactions between adipocytes and immune cells in WAT, mediated by lipokines and cytokines/adipokines and metabolites, are essential for the healthy functioning of WAT ([21,22,23,24]; reviewed in [25,26]). Also, the proliferation and differentiation of stem cells and preadipocytes depends on the local niche provided by both the endothelial mural cell compartment [11,27] and macrophages [28]. Also these processes are mainly coordinated by the autocrine and paracrine effects of the WAT-borne signalling molecules and metabolites [29,30]. Therefore, the immunometabolism [31] of WAT, i.e., the cross talk between cells of the immune system contained in the tissue and the tissue metabolism (see above and [26]) contributes to either a lean or obese phenotype. These opposite systemic effects reflect either enhancing or lowering the capacity of WAT for buffering circulating fatty acids. Hence, both the amount of WAT and its immunometabolic properties represent a therapeutic target for the treatment of obesity and associated diseases (reviewed in [25,26,32]). 

Our previous studies have shown that the induction of obesity and deterioration of the immunometabolism of WAT in mice fed an obesogenic high-fat diet could be ameliorated in response to dietary supplementation with long-chain polyunsaturated fatty acids of the n-3 series (omega-3 PUFA; reviewed in [26,32]), namely eicosapentaenoic acid (EPA; 20:5 ω-3) and docosahexaenoic acid (DHA; 22:6 ω-3). The effects of omega-3 PUFA were even more pronounced when a combined intervention with either calorie restriction [33] or antidiabetic drugs was used [34]. The multiple beneficial effects on health, exerted by omega-3 PUFA, are mediated by these PUFA themselves, related lipid mediators, and multiple intracellular signalling pathways (reviewed by us in [25,26,32,35]). 

Our previous results also indicated that, in addition to modulating the immunometabolic properties of WAT, the reduced accumulation of body fat due to omega-3 PUFA supplementation in mice fed a high-fat diet was in part due to a prevention of the increase in tissue cell number [36,37]. Therefore, the main goal of this study was to characterize in detail the cell types involved the abolishment of hyperplastic growth of WAT in mice fed a high-fat diet in response to the omega-3 PUFA supplementation. 

## 2. Results

### 2.1. Effect of Omega-3 PUFA on Body Weight and eWAT

C57BL/6N male mice were fed either a standard (STD) or high-fat (HFD) diet or a high-fat diet supplemented with omega-3 PUFA (HFF) for one or eight weeks starting at 13 weeks of age. Both the HFD and HFF diet increased the body weight and eWAT weight at both Week 1 and Week 8 compared to the STD diet, with no impact of omega-3 PUFA on body weight compared to HFD (Table 1). However, eWAT weight tended to be lower after Week 1 and was reduced by 20% at Week 8 in the HFF compared to the HFD fed mice (Table 1). 

The DNA content of eWAT, a surrogate marker of cell number in the tissue, was higher at Week 8 than Week 1. It increased much faster in the mice fed the high-fat diet, resulting in a 3.0-fold and 2.5-fold higher DNA content in the HFD and HFF mice, respectively, compared to the STD mice at Week 8 (Table 1). Thus, omega-3 PUFA supplementation abolished in part the increase in eWAT cell number in the mice fed a high-fat diet, with a significant effect (1.2-fold difference between the HFD and HFF mice) at Week 8. The trend for this effect was already apparent at Week 1 (Table 1). The differences in eWAT DNA content at Week 8 were mirrored by those in the total DNA content of the fraction of collagenase-liberated adipocytes from eWAT. However, in this case, only a trend to reduce DNA content by omega-3 PUFA was observed (Table 1).

**Figure 1:**
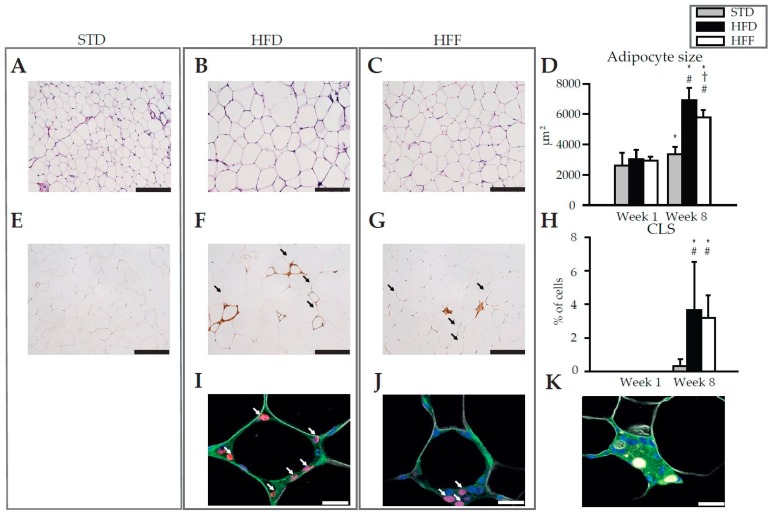
Morphology and immunohistochemistry of eWAT. Representative histological sections of eWAT from mice fed STD (**A**,**E**), HFD (**B**,**F**,**I**) or HFF (**C**,**G**,**J**,**K**) diet at Week 8. Hematoxylin and eosin staining for morphometry of adipocytes (**A**,**B**,**C**), as evaluated in (**D**). Immunohistochemical staining using macrophage marker MAC2 for quantification of CLS (**E**,**F**,**G**; arrows), as evaluated in (**H**). Representative sections showing the detection of macrophage proliferation within CLS based on immunofluorescence staining (**I**,**J**; nuclei by DAPI, blue; macrophages by anti-F4/80, green; surface of lipid droplets by anti-perilipin 1, white; proliferating nuclei by anti-Ki67, red; proliferating macrophages are indicated with arrows). Representative section showing multinucleated giant cells (**K**). Data are means ± SD; *n* = 6–8. ^*^ Significant difference from Week 1 between mice on same diets; ^†^ significant difference from HFD for the same period of dietary intervention; ^#^ significant difference from STD for the same period of dietary intervention. Bar represents 200 μm (**A**,**B**,**C**,**E**,**F**,**G**) or 20 μm (**I**,**J**,**K**). For morphology and immunohistochemistry of eWAT at Week 1, see Supplementary Figure S1.

Histological examination and morphometry of adipocytes in eWAT revealed that adipocyte size increased between Week 1 and Week 8 in both the HFD and HFF mice. This increase was less pronounced in the HFF mice (Figure 1A–D). In the STD mice, adipocyte size also increased between Week 1 and Week 8, however, this increase was very small (see Supplementary Figure S1A–C for the hematoxylin and eosin staining of eWAT sections at Week 1). At Week 8 in both the HFD (Figure 1F) and HFF (Figure 1G) mice, but not in the STD mice (Figure 1E), the immunostaining of eWAT revealed crown-like structures (CLS) that are formed by macrophages aggregated around dying adipocytes [38]. However, the abundance of CLS was not influenced by omega-3 PUFA supplementation (Figure 1F,G,H). At Week 1, no CLS were detected in any of the dietary groups (Supplementary Figure S1D–F). Also the proliferation of macrophages within CLS, assessed immunohistochemically using Ki67 staining (Ki67 is a commonly used marker of proliferating nuclei, which was shown to control heterochromatin organisation, see [39,40]) did not differ between the dietary groups (HFD: 19 ± 3% vs. HFF: 16 ± 1% of all CLS-contained nuclei; Figure 1I,J). Interestingly, multinucleated giant cells (MGCs) were only observed in the HFF mice (Figure 1K), suggesting macrophage fusion [41,42]. 

These results documented that the increase in eWAT weight in response to the high-fat diet emerged from both tissue hypertrophy and hyperplasia, and that both these processes could be partially counteracted by omega-3 PUFA supplementation. Moreover, the results suggested that the effect of omega-3 PUFA on tissue cell number probably does not include changes in tissue macrophage content.

### 2.2. Anti-Inflammatory Effects of Omega-3 PUFA in eWAT in Mice Fed High-Fat Diet

**Figure 2:**
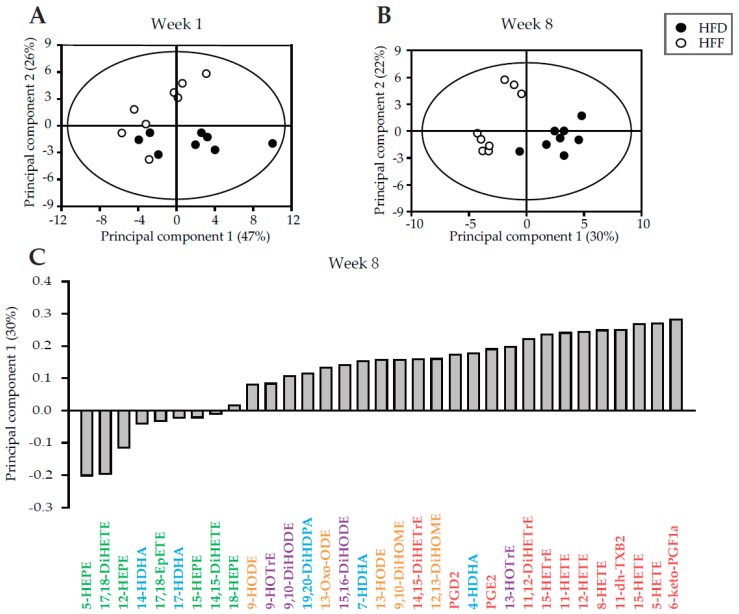
Principal component analysis of the lipidomic data from eWAT of the HFD and HFF mice. Score plots of the principal components 1 and 2 were generated using the lipid mediator profiles at Week 1 (**A**) and Week 8 (**B**). At Week 8 (**C**), results were expressed as a contribution score plot showing one bar per variable, indicating which species differ most between the groups and in which direction. Lipids derived from AA (red), LA (orange), ALA (purple), DHA (blue), and EPA (green) are discerned by colors. For the source data and the abbreviations, see Supplementary Table S1.

The lack of effect of omega-3 PUFA on the CLS macrophages content in eWAT, despite the decrease in eWAT cell number, prompted us to verify the expected anti-inflammatory effect of omega-3 PUFA in this tissue. First, we addressed the effect on the formation of related lipid mediators [25,43] in eWAT. In the HFD and HFF mice, in total 33 metabolites of arachidonoic acid (AA), α-linolenic acid (ALA), dihomo-γ-linolenic acid (DGLA), DHA, EPA, and linoleic acid (LA) were evaluated at both Week 1 and Week 8 (see Supplementary Table S1). Principal component analysis (PCA) of the data was used to obtain a global view on the effects of the diet and the duration of the dietary intervention. Separation of the dietary groups was evident at both time points, and it was more robust at Week 8 (Figure 2A,B). Among the most discriminating analytes were: hydroxyderivatives of AA, prostaglandins, thromboxane, and hydroxyderivatives of EPA (Figure 2C). As for the levels of individual fatty acid metabolites (Supplementary Table S1), either at Week 1 or Week 8, AA-, ALA-, DGLA-, and LA-derived metabolites were mostly lower in the HFF mice than in the HFD mice, while the levels of EPA-derived lipid mediators in the HFF mice were relatively high. In contrast, the levels of DHA-derived lipid mediators were similar in both dietary groups at both Week 1 and Week 8. Therefore, the pattern of changes in lipid mediators showed an expected shift from the AA-derived pro-inflammatory toward the EPA-derived anti-inflammatory mediators in response to omega-3 PUFA supplementation.

Since the hydroxyderivatives of AA and EPA are metabolic products of lipoxygenases (LOX), the expression of the genes for various types of LOX (*Alox5*, *Alox12*, and *Alox15*) was quantified using real-time quantitative PCR (qPCR). However, no differences in the expression of the above genes between the dietary groups were observed in the whole eWAT nor in the subfractions of cells isolated from eWAT, with no dependence on the duration of the dietary intervention (Supplementary Table S2). Tissue levels of lipid mediators are not only influenced by the rate of synthesis, but also by the rate of degradation of these mediators. Importantly, down-regulation of the expression of 15-hydroxyprostaglandin dehydrogenase (*15-Pgdh*) in response to omega-3 PUFA supplementation was detected in both the stromal-vascular fraction (SVF) and adipocyte fraction isolated from eWAT at Week 8 (Supplementary Table S2). This enzyme is responsible for the inactivation of selected prostaglandins, leukotrienes, and several hydroxy-eicosatetraenoic acid species (HETEs; reviewed in [44]).

**Figure 3:**
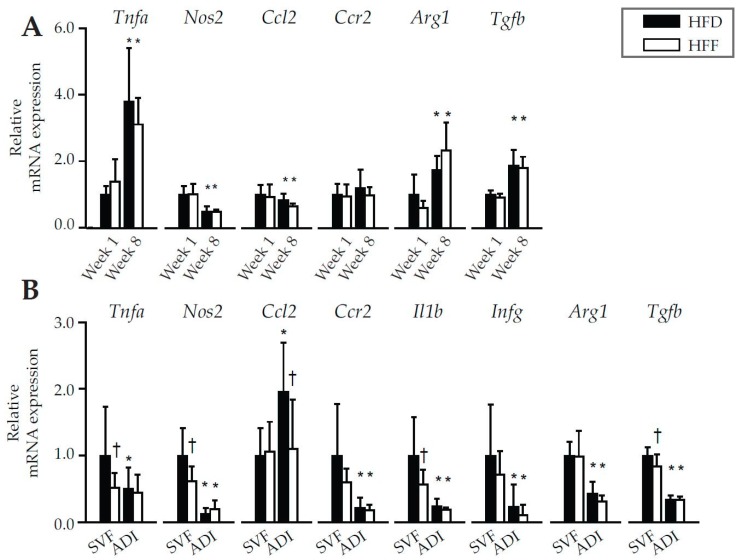
Gene expression of pro- and anti-inflammatory markers in eWAT of mice fed HFD or HFF diet for 1 or 8 weeks (**A**), or in stromal-vascular fraction (SVF) cells or adipocytes (ADI) isolated from eWAT of mice fed the respective diets for 8 weeks (**B**). Data were normalized to the geometrical mean of two reference genes, *Hprt* and *EF1α* in (**A**), and *EF1α* and *Rn18s* in (**B**), and expressed relative to the HFD mice at Week 1 for whole eWAT depot (**A**) or to SVF of HFD group (Week 8) for SVF and ADI (**B**). Data are means ± SD; *n* = 6–8. ^*^ Significant difference from Week 1 between mice with the same diet; ^†^ significant difference for the same period of dietary intervention.

Subsequently, inflammatory gene expression profiles in eWAT of the HFD and HFF mice were compared. This analysis did not reveal any differences between the dietary groups, neither at Week 1 nor at Week 8 (Figure 3A). Therefore, the analysis was repeated using both SVF and adipocytes isolated from eWAT at Week 8 (Figure 3B). Expression of most of the evaluated inflammatory markers, such as tumor necrosis factor (*Tnfa*), nitric oxide synthase 2 (*Nos2*), C-C chemokine receptor type 2 (*Ccr2*), interleukin 1 beta (*Il1b*), and interferon gamma (*Ifng*) as well as transforming growth factor beta (*Tgfb*; regulator of inflammatory processes), was (or tended to be) lower in the SVF of the HFF mice. In the adipocyte fraction, expression of the above genes was not affected by the omega-3 PUFA supplementation, while the expression of chemokine (C-C motif) ligand 2 (*Ccl2*), another inflammatory marker, was the only one to be down-regulated by omega-3 PUFA in the adipocyte fraction (Figure 3B). The expression of the anti-inflammatory marker arginase (*Arg1*) did not differ between the dietary groups, either in eWAT (Figure 3A) or in the isolated cells (Figure 3B). Thus, mRNA analysis confirmed the lower pro-inflammatory profile of SVF cells within eWAT in HFF compared to the HFD mice. 

### 2.3. Changes in Immune Cell Abundance Cannot Explain the Effect of Omega-3 PUFA on eWAT Cell Number in Mice Fed High-Fat Diet

**Figure 4:**
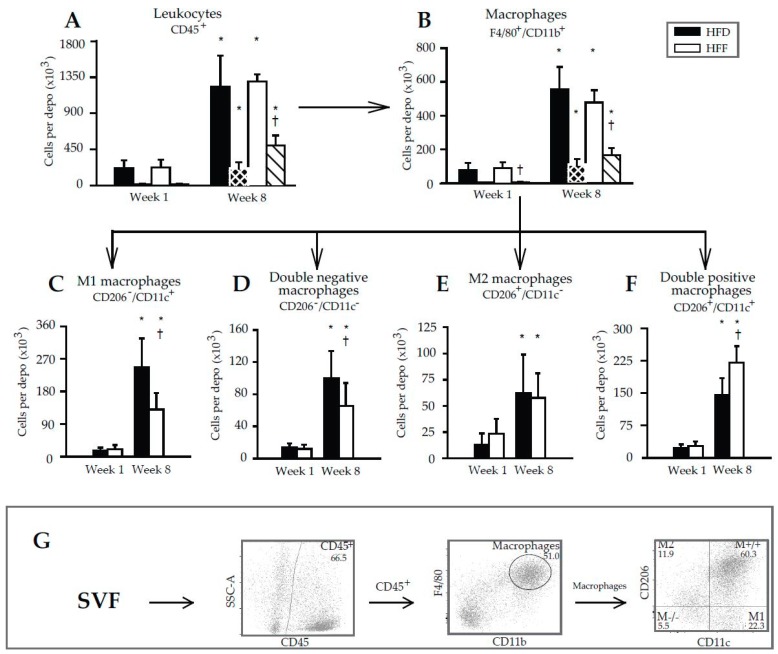
Flow cytometry analysis of immune cell subsets in SVF isolated from eWAT. Numbers of cells are calculated per depot. Arrows indicate the gating strategy used. Cells were first gated on size and singularity for further analysis. Single cells were gated based on the expression of CD45 to identify leukocytes (**A**). Leukocytes were then gated on the co-expression of CD11b and F4/80 to identify macrophages (**B**). Finally, macrophages were further subdivided based on the expression of CD206 and CD11c into: M1 macrophages (CD206^−^/CD11c^+^; **C**), double-negative macrophages (CD206^−^/CD11c^−^; **D**), M2 macrophages (CD206^+^/CD11c^−^; **E**) and double-positive macrophages (CD206^+^/CD11c^+^; **F**). Striped columns (black with white stripes, HFD; white with black stripes, HFF) show the amount of proliferating cells per depot, which were detected using antibodies specific for the Ki67 proliferation marker. Illustrative flow cytometry plots and gating strategy are also shown (**G**). Data are means ± SD.; *n* = 6–8. ^*^ Significant difference compared to Week 1 with the same diets; ^†^ significant difference between the diets for the same period of dietary intervention.

The above results documented the anti-inflammatory effect of omega-3 PUFA in eWAT and prompted us to characterize in detail the immune cells in the tissue. Flow cytometry was performed using SVF cells isolated from eWAT (Figure 4A–F; for illustrative flow cytometry plots and gating strategy—see Figure 4G) and the appropriate panel of antibodies (Supplementary Tables S3 and S4). The number of leukocytes in the whole eWAT depot (CD45^+^ cell population containing the majority of the WAT immune cells [45]) increased 5.6- and 5.7-fold in the HFD and HFF mice, respectively, between Week 1 and Week 8. It was similar in both dietary groups at any given time (Figure 4A). The corresponding increases in the number of eWAT macrophages (i.e., a subfraction of the leukocyte population characterized as CD45^+^/F4/80^+^/CD11b^+^ cells) was ~7.4- (HFD) and ~5.3-fold (HFF); again, the cell number was not significantly affected by the diet (Figure 4B). However, the omega-3 PUFA supplementation tended to limit the high-fat diet induced increase in eWAT macrophage content (Figure 4B). 

The proliferation of both leukocytes and macrophages in the SVF, assessed using Ki67 antibodies (Figure 4A,B; striped columns), was negligible at Week 1 but it was relatively high at Week 8. This induction was stronger in the HFF than the HFD mice, resulting in a significantly higher fraction of the proliferating cells in the total population of leukocytes and macrophages at Week 8 (Supplementary Figure S2).

**Figure 5:**
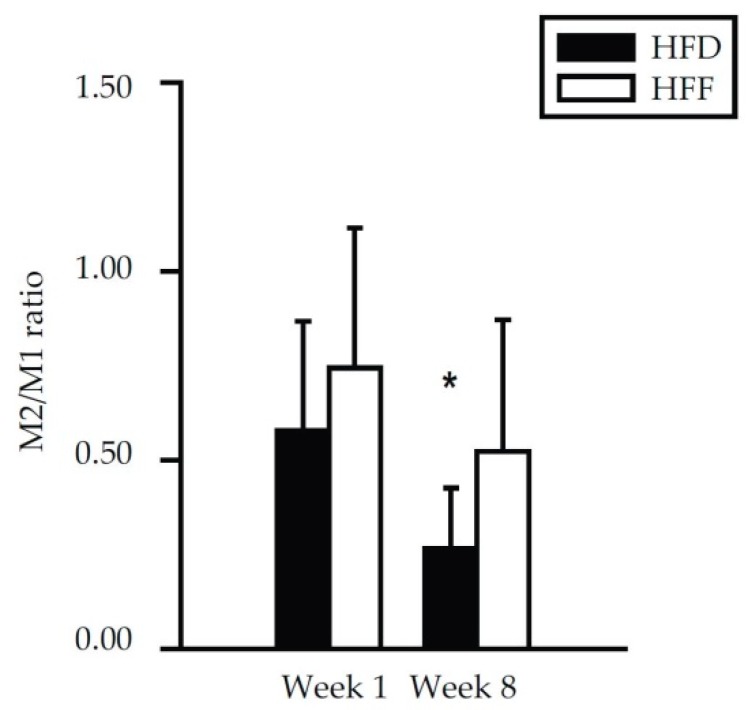
Ratio between M2 and M1 macrophages in eWAT of mice fed HFD or HFF diet for 1 week or 8 weeks as determined by flow cytometry. Data from Figure 4C,E were re-plotted. ^*^ Significant difference compared to Week 1 within the diets.

Further subfractionation of the macrophage population was performed using the CD206 and CD11c markers, resulting in the detection of four different macrophage subtypes (Figure 4C–F). At Week 1, no differences between the dietary groups were found. At Week 8, the eWAT content of both CD206^−^/CD11c^+^ (M1; Figure 4C) and CD206^−^/CD11c^−^ (double negative; Figure 4D) macrophages was relatively low in the HFF mice. The eWAT content of CD206^+^/CD11c^−^ (M2; Figure 4E) macrophages did not differ between the subgroups, but CD206^+^/CD11c^+^ (double positive; Figure 4F) macrophages were more abundant in the HFF mice. The proportion in the content of M2 and M1 macrophages (i.e., the M2/M1 ratio; Figure 5) decreased between Week 1 and Week 8 in the HFD and tended to decrease in the HFF mice. The M2/M1 ratio tended to be higher in the HFF mice and this difference tended to increase with the duration of the dietary intervention. Both the relatively low number of M1 macrophages in the HFF mice at Week 8, and the relatively high number of double positive macrophages in these mice are consistent with the anti-inflammatory effect of the omega-3 PUFA supplementation [20,46].

Thus, both qPCR and flow cytometry analysis revealed the lower pro-inflammatory profile of immune cells within eWAT in the HFF compared to the HFD mice at Week 8. However, neither the total number of macrophages nor the numbers of other cells in the leukocyte population, could explain the lower number of eWAT cells in the HFF mice at Week 8 (see the DNA content in Table 1). 

### 2.4. Limited Increase in Adipose Lineage and Endothelial Cells Numbers in eWAT of Mice Fed High-Fat Diet in Response to Omega-3 PUFA

**Figure 6:**
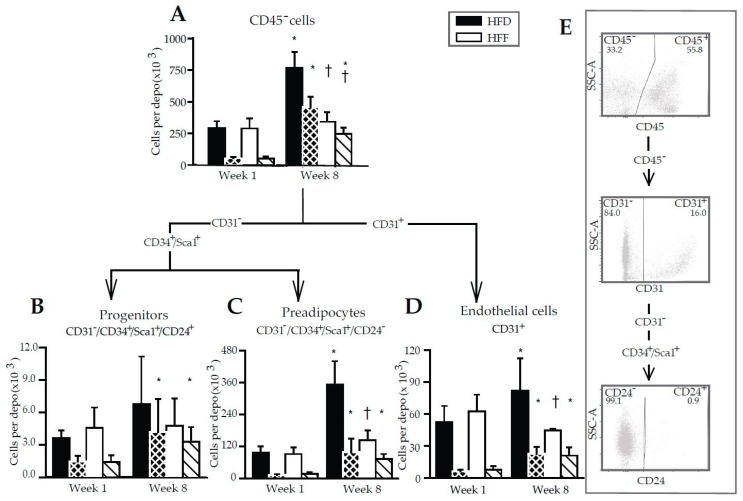
Flow cytometry analysis of non-immune cell subsets in SVF isolated from eWAT. The numbers of cells are calculated per depot. Arrows indicate the gating strategy used. Cells were first gated on size and singularity for further analysis. Single cells were gated on the lack of CD45 expression to identify non-lymfoid cells (**A**). CD45^−^ cells were then gated on CD31 expression to identify endothelial cells (**D**). CD31^−^ cells were further subdivided based on the expression of Sca1, CD34 and CD24 into progenitors (CD34^+^/Sca1^+^/CD24^+^; **B**) and preadipocytes (CD34^+^/Sca1^+^/CD24^−^; **C**). Striped columns (black with white stripes, HFD; white with black stripes, HFF) show the amount of proliferating cells per depot, which were detected using antibodies specific for the Ki67 proliferation marker. Illustrative flow cytometry plots and gating strategy are also shown (**E**). Data are means ± SD; *n* = 5–6. ^*^ Significant difference compared to Week 1 for mice fed the same diet; ^†^ significant difference between the diets for the same period of dietary intervention.

Changes in the immune cells number could not explain the abolishment of eWAT hyperplastic growth by omega-3 PUFA (although the changes in the composition of the immune cells could be important for this mechanism, see Discussion). Therefore, the CD45^−^ cells contained in the SVF, i.e., the adipose lineage and endothelial cells [45], were characterized (Figure 6A–D; for illustrative flow cytometry plots and the gating strategy, see Figure 6E). In the HFD mice, the number of these cells (recalculated per the whole eWAT depot) was similar in the HFD and HFF mice at Week 1, while in the HFD mice, it increased ~2.7-fold between Week 1 and Week 8. The corresponding increase in the HFF mice was only ~1.2-fold, resulting in a ~2.2-fold lower number of CD45^−^ cells in the eWAT of the HFF compared to the HFD mice at Week 8 (Figure 6A). The proliferation of CD45^−^ cells (Figure 6A, striped columns; Supplementary Figure S3A) was similar in both dietary groups at Week 1, and it increased ~8.8-fold (HFD) and ~5.1-fold (HFF) between Week 1 and Week 8. Thus, at Week 8, the fraction of the proliferating cells in the total CD45^−^ cell population was higher in the HFF than in the HFD mice (Figure 6A; Supplementary Figure S3A).

Further analysis of the CD45^−^ cell population using the CD31, CD34, Sca1, and CD24 markers enabled the adipose progenitor cells (Figure 6B), preadipocytes (Figure 6C) and endothelial cells (Figure 6D) to be characterized. The eWAT quantity of progenitors was similar in all the groups, irrespective of the duration of high-fat feeding or the omega-3 PUFA supplementation, while the proliferation of these cells increased ~2.8-fold (HFD) and ~2.3-fold (HFF) between Week 1 and Week 8 (Figure 6B; Supplementary Figure S3B). 

The eWAT number of preadipocytes did not differ between the HFD and HFF mice at Week 1, and increased much more in the HFD (~3.7- fold) compared with the HFF (~1.6-fold) mice between Week 1 and Week 8, resulting in ~2.5-fold lower number of preadipocytes in the HFF mice compared to the HFD mice at Week 8 (Figure 6C). This was in agreement with the expression of several markers of adipogenesis and mainly of preadipocyte factor 1 (*Pref1*, a marker of preadipocytes and inhibitor of adipocyte differentiation [47]), which was relatively low in both the eWAT and SVF of the HFF mice at Week 8, but was similar in adipocytes isolated from the eWAT of both dietary groups (Supplementary Table S5). Also, the expression of the genes for peroxisome proliferator-activated receptor γ (*Pparg;* a late adipogenic marker; [9]) and platelet-derived growth factor receptor β (*Pdgfrb*; expressed mainly in adipocyte progenitor cells [48]) was relatively low in the SVF isolated from the eWAT of the HFF mice at Week 8 (Supplementary Table S5). The proliferation of preadipocytes was increased in response to high-fat feeding, resulting in a ~7.7-fold (HFD) and ~4.2-fold (HFF) elevation between Week 1 and Week 8 (Figure 6C; Supplementary Figure S3C). 

The number of eWAT endothelial cells was similar in all groups, except for Week 8, when the number of these cells was ~1.8-fold lower in the HFF than the HFD mice (Figure 6D). The proliferation of endothelial cells was increased in response to high-fat feeding, i.e., exhibiting a ~3.7-fold (HFD) and ~2.7-fold (HFF) increase between Week 1 and Week 8 (Figure 6D; Supplementary Figure S3D). 

Collectively (see also Supplementary Figure S4), the above results documented that counteracting the HFD-driven increase in eWAT cell number by omega-3 PUFA supplementation did not reflect a change in the number of leukocytes. On the contrary, the abolishment of eWAT hyperplasia reflected selective changes in the development of the adipose lineage and endothelial cells, especially in terms of abolishing the increase in the number of preadipocytes and the decrease in the number of endothelial cells. The number of proliferating cells in all the identified classes of CD45^−^ cells did not differ between the dietary groups (Figure 6A–D); however, when expressed as a percentage, the proportion of proliferating cells was higher in the HFF than the HFD mice at Week 8 (Supplementary Figure S3).

## 3. Discussion

This study was focused on the mechanism by which omega-3 PUFA limit the accumulation of WAT in mice fed an obesogenic high-fat diet. We confirmed the previous results documenting that both hypertrophy and hyperplasia of eWAT were partially counteracted by the omega-3 PUFA supplementation of the diet (reviewed in [26,32]). The principal new finding was that while the hyperplastic growth of the tissue resulted from the increased number of leukocytes, as well as the adipose lineage and endothelial cells, the amelioration of eWAT hyperplasia by omega-3 PUFA was reflected in the reduced numbers of preadipocytes and adipocytes as well as endothelial cells, but not the immune cells. 

Our results support the view that the activity of the immune cells in WAT and tissue metabolism are closely connected ([31]). They suggest that both (i) increased levels of EPA and DHA, and (ii) changing pattern of formation of lipid mediators and cytokines/adipokines in WAT, in response to the omega-3 PUFA supplementation, could trigger the counteraction of the diet-induced hypertrophy and hyperplasia of the tissue. Specifically the time-dependent induction of the anti-inflammatory 5-HEPE and 17,18-diHETE, in the face of the decreased content of pro-inflammatory HETEs (5-HETE, 8-HETE, 12-HETE and 15-HETE), was probably involved (for the biochemical pathways of the formation of these lipid mediators, see reviews [25,26]). Accordingly, a reduced expression of the genes for pro-inflammatory enzyme (*Nos2*) and cytokines (*Tnfa*, *Tgfb* and *Il1b*) was also observed (for more information about these markers, see [29,30]). 

Macrophages, adipocytes, as well as other cells contained in WAT represent the source of the above signalling molecules. However, precise identification of the cells involved is not known and was outside the scope of this study. The observed changes in the abundancy of the four subtypes of WAT macrophages in response to the omega-3 PUFA supplementation, observed at Week 8, were in general agreement with the involvement of these cells in the change in WAT inflammatory status and the secretion of lipid mediators and adipokines. Thus, the content of the pro-inflammatory M1 macrophages decreased. The double-positive (mixed M1/M2) macrophage content was higher in the HFF than in the HFD mice, in accordance with the possible involvement of these cells in the resolution of WAT inflammation [46] as well as relatively high activity of lipid catabolism in these cells [49], which is linked with the anti-inflammatory macrophage phenotype (reviewed in [25]). A decrease in the content of double-negative macrophages in response to the omega-3 PUFA supplementation was also observed. However, the role of these cells in inflammatory status remains poorly defined. In accordance with the previous study [50], MGCs were detected in the HFF mice at Week 8. MGCs are formed by the fusion of M2 or double-positive macrophages [41], with the participation of scavenger receptor CD36 [51]. These cells have an enhanced reactive oxygen species generating capacity [41] and are engaged in tissue remodeling and repair [42]. Therefore, MGCs probably contributed to tissue remodeling in response to omega-3 PUFA.

Regarding the limitation of the high-fat-diet-induced WAT hypertrophy by the omega-3 PUFA, it should be stressed that this effect was independent of food consumption [33,52]. On the other hand, the induction of fatty acid oxidation in response to omega-3 PUFA was observed in the liver [53], intestine [54], muscle [55,56], and possibly also in brown fat and other tissues (reviewed by us in [25,26,32,35]). In particular, modulation of the cross talk between WAT and liver metabolism could play an important role in the counteraction of eWAT hypertrophy [25,57]. The above metabolic effects of omega-3 PUFA are mediated by (i) these PUFA themselves, (ii) their bioactive metabolites—lipid mediators, or (iii) suppression of tissue levels of endocannabinoids; all these potential mechanisms operate also in WAT (reviewed in [25,26,35]). Multiple receptors and intracellular signalling pathways involved in the modification of WAT immunometabolism by omega-3 PUFA and related lipid mediators (see above) have been identified (reviewed in [26,32]; see Figure 1 of ref. [25]). 

Regarding the limitation of the high-fat diet-induced WAT hyperplasia by the omega-3 PUFA, it should be stressed that especially in rodents, WAT exerts a high potential for hyperplastic growth (see [8,13,16,17,18]). Therefore, the mouse model is instrumental to characterize this omega-3 PUFA effect. This WAT hyperplastic remodeling can be very quick [8,16], as it includes the proliferation of immune cells [13,34,58,59] as well as adipocyte progenitor cells and their differentiation into preadipocytes [8,16,17,60,61]. As for the eWAT immune cells studied here, the strong elevation in the leukocytes (including macrophages) content in response to high-fat feeding was documented using both flow cytometry and immunostaining of the CLS-contained macrophages. Both approaches revealed a trend for a lower macrophage content in the HFF than in the HFD mice at Week 8, but the differences were not significant. These results were consistent with some of the previous studies in mice from our laboratory [33,62] and by others [50]. They are also in agreement with some human studies [63,64], showing no effect of omega-3 PUFA on WAT macrophage content [33,62]. On the other hand, the reduction of WAT macrophage content by omega-3 PUFA was also observed before, both in dietary obese mice [33,34,65,66] and human subjects with insulin resistance [67]. Due to the many variables involved, the reasons for this discrepancy remain unknown. 

As for the adipose lineage cells contained in the CD45^−^ population [45] studied here (except for the mature adipocytes), the expected increase in the number of these cells in response to high-fat feeding was observed (flow cytometry data). Supplementation of the diet with omega-3 PUFA completely eliminated the induction of the CD45^−^ cells population. The effect on the CD45^−^ cells population especially reflected the compromised increase in the number of preadipocytes. The immune cells were not involved in the reduction of eWAT cells number by omega-3 PUFA. However, these cells could interact with the adipose lineage cells and contribute to this limitation. For instance, M2 macrophages inhibit the proliferation of adipogenic precursors through the CD206/TGFb signalling pathway [68]. Also 9- and 13-HODE (see Figure 2) are involved in the communication of macrophages with preadipocytes [69].

Furthermore, also a reduction in the endothelial cells content in eWAT in response to the omega-3 PUFA supplementation was observed at Week 8, despite the number of endothelial cells was not affected by the HFD feeding. It has been published that angiogenesis was regulated by PUFA at least in part through the action of the prostanoids (suppressed by prostaglandin E3 and augmented by prostaglandin E2; [70]). Therefore, in the reduction of endothelial cells number, the observed decrease in prostaglandin E2 levels could be involved. Moreover, 12/15 LOX is associated with monocyte-endothelial interaction [71]; indeed lower levels of 12-HETE in the HFF as compared to HFD mice were found (see Supplementary Table S1).

The limitation of this study is the lack of quantitative information regarding the adipocytes abundance in eWAT, however, DNA content measurement in the collagenase-liberated adipocytes from eWAT suggested that mature adipocytes contributed to both (i) the increase in eWAT cell number induced by a high-fat diet, and (ii) the amelioration of this process by omega-3 PUFA supplementation. 

Since the proportion of proliferating non-immune cells (including the progenitors and preadipocytes) as well as endothelial cells in the whole population of these cells was higher at Week 8 in the HFF than in the HFD mice, while the eWAT content of these cells was lower in the HFF mice, it is to be inferred that the omega-3 PUFA supplementation likely resulted in increased removal/turnover of the above cell types in the eWAT of the obese mice. Indeed, previous studies described the induction of the apoptosis of preadipocytes/adipocytes by omega-3 PUFA, both in vivo in the WAT of mice [72] and *in vitro* in adipocytes differentiating in cell culture [73,74]. In addition, omega-3 PUFA-derived lipid mediators were shown to be involved in the regulation of apoptosis, e.g., with the products of 5- and 12-LOX (5-HETE and 12-HETE) exerting an inhibitory effect *in vitro* [72,75]. This is in agreement with our lipidomic data at Week 8, showing that: (i) 5-HETE and 12-HETE belonged to the most discriminating analytes between the HFD and HFF mice; and (ii) the levels of both HETEs were relatively low in the HFF mice (see Supplementary Table S1).

In this study, we focused on eWAT, the typical WAT depot forming the majority of abdominal fat in dietary obese mice [33,35,62,76]. However, our previous study documented that the hyperplastic growth of subcutaneous WAT in the mice was also compromised by the omega-3 PUFA supplementation, although a relatively high dose of omega-3 PUFA was required [36]. These results suggested that omega-3 PUFA could reduce the hyperplastic growth of all WAT depots. Fat-depot-specific differences in sensitivity to omega-3 PUFA may exist. That the effect is highly pronounced in WAT in the abdomen may be of practical importance. It has been known for a long time that the accumulation of visceral fat, which characterizes upper body obesity, correlates with metabolic syndrome [77]. 

This study helps to better understand the mechanism behind the prevention of obesity development by omega-3 PUFA, which is frequently observed in animal models (see our previous studies [33,34,36,52,78], and studies by others; reviewed in [25]) but is of only marginal significance in humans (reviewed in [25]). There may be multiple reasons for this discrepancy, including the dose of omega-3 [35], the effect of the composition of the bulk of dietary lipids [62], possible inter-species differences in intracellular signalling and the formation of lipid mediators involved in the effects of omega-3 PUFA, and others. Whether the reversal of obesity in response to omega-3 PUFA in mice [30,66,76] also reflects in part the reduction of tissue cell number remains to be established. 

In conclusion, our results provide evidence for the involvement of omega-3 PUFA in the remodelling of WAT in mice fed a high-fat diet. Changes in macrophages polarization, reflected by their morphology and metabolism, but not the number of immune cells *per se*, contributed to the remodelling process. Prevention of the diet-induced hyperplasia of WAT by omega-3 PUFA could be explained by a limited increase in the number of adipose lineage cells, as well as by the decrease in the number of endothelial cells. To the best of our knowledge, to date the prevention of diet-induced hyperplasia of WAT by omega-3 PUFA is only documented by this and two previous studies from our laboratory [36,37]. Our results support the notion [7,37] that adiposity is closely linked to the control of fat cell turnover and that there could be mechanisms that control fat cell proliferation independently of energy balance.

## 4. Materials and Methods 

### 4.1. Animals

Male mice (C57BL/6N, Charles River, Germany) were fed the STD diet (3.4% wt/wt as lipids; extruded ssniff R/M-H from Ssniff Spezialdiaten GmbH, Soest, Germany) from the moment they were brought to the institute´s animal house at the age of 6 weeks and maintained on a 12-h light/dark cycle (light from 6:00 a.m.) at 22 °C. At 13 weeks of age, mice were randomly divided into three groups that were subjected to various dietary interventions (i) the STD diet, (ii) a corn oil-based high-fat diet (HFD diet; ~35% wt/wt as lipids; or (iii) an HFD-based diet, in which 15% (wt/wt) of dietary lipids (corn oil) was replaced with the EPA + DHA triglyceride concentrate Epax 1050 TG (HFF diet; Epax 1050 TG contained ~14% EPA and ~46% DHA, wt/wt; EPAX AS, Aalesund, Norway) to achieve a total EPA + DHA concentration of ~30 g/kg diet. The numbers of animals are specified in each caption. For the macronutrient and fatty acid composition of the diets, see ESM Tables 1 and 2 of [34] (the HFD and HFF diets are identical with the cHF and cHF + F diets, respectively). The omega-6/omega-3 ratio based on the fatty acid composition of the diets is 26.5 for HFD and 3.5 for HFF, respectively (see [34]). Both high-fat diets were prepared at the Institute of Physiology in Prague (Prague, Czech Republic) as described previously (see ESM Table 1 of [34]). These diets have already been used in several studies [33,34,35,36,37,43,54,55,62,76]

Two dietary intervention experiments (see above) were performed lasting either 1 or 8 weeks. Animals were killed under ether anesthesia in a random-fed state (between 8:00 and 10:00 a.m.). Body weight and food consumption were recorded daily during Week 1, and then weekly during the later stages of the differential dietary treatment. eWAT was collected and either flash frozen and stored in liquid nitrogen or processed for ex vivo biochemical analyses. All animal procedures were conducted in accordance with all appropriate regulatory standards under protocol 81/2016 (approval date: 2016-10-18) approved by Animal Care and Use Committee of the Czech Academy of Sciences and followed the guidelines for the use and care of laboratory animals of the Institute of Physiology.

### 4.2. RNA Isolation and Gene Expression Analysis

Total RNA was isolated from eWAT (~100 mg) using TRI Reagent (Sigma-Aldrich, Prague, Czech Republic, see [33]). qPCR and a LightCycler480 (Roche, Prague, Czech Republic) were used to determine the mRNA levels of various transcripts. Data were normalized to the geometrical mean of two reference genes hypoxanthine guanine phosphoribosyl transferase (*Hprt*) and eukaryotic translation elongation factor 1 alpha 1 *(EF1a*) for the analysis using whole eWAT samples and *EF1α* and 18S ribosomal RNA (*Rn18s*) for the analysis using SVF and adipocyte fractions; see Supplementary Table S6 for PCR primer sequences.

### 4.3. Flow Cytometry

eWAT was enzymatically digested in Krebs-Ringer Bicarbonate Buffer (KRB; pH = 7.4) containing 0.1% collagenase type (Sigma-Aldrich, Munich, Germany) and 4% BSA for 30 min at 37 °C. Released cells were spun down (500× *g*, 10 min, 4 °C) to separate SVF cells from floating adipocytes. SVF cells were resuspended in KRB containing 10 mM EDTA and 4% BSA, and passed through a 42 µm filter. Red blood cells were lysed using the Lysis Buffer (eBioscience, San Diego, CA, USA). Cells were incubated in Fc block solution for the prevention of non-specific binding (AntiMouse CD16/CD32; eBioScience, San Diego, CA, USA) for 10 min on ice and stained with the indicated antibodies against extracellular markers for 30 min at 4 °C (see Supplementary Tables S3 and S4 for the antibodies). Before intracellular staining, the cells were fixed and permeabilized (Intracellular Fix and Perm Set, eBioScience, San Diego, CA, USA), and then stained with Ki67 antibody for 30 min at 4 °C. After washing, the stained cells were analyzed using a BD LSR II flow cytometer (BD Biosciences, San Jose, CA, USA). Data were analyzed using FlowJo 10.2 software (Tree Star, Ashland, OR, USA).

### 4.4. Analysis of Lipid Mediators 

Lipid mediators were purified from eWAT samples (~100 mg), flash-frozen and stored in liquid nitrogen after dissection, using solid phase extraction procedure as before [35]. Analysis was performed using a UPLC-MS/MS platform (Ultimate 3000 RSLC, Dionex/Thermo and QTRAP 5500, AB SCIEX, Framingham, MA, USA) equipped with a C18 column 150 cm × 2.1 cm, 1.9 µm with a precolumn. Analytes were ionized in negative ion mode and detected with a multiple reaction monitoring method as before [24,35].

### 4.5. DNA Measurement

DNA was estimated fluorometrically using Hoechst 3328 in tissue and adipocytes samples digested with proteinase K as before [79]. 

### 4.6. Histological and Immunohistochemical Analysis of eWAT

These analyses were performed similarly as described previously [28]. Formalin-fixed paraffin-embedded sections (5 μm) were stained using hematoxylin–eosin for the morphometry of adipocytes, or processed by immunohistochemistry or immunofluorescence. The morphometry data are based on approximately 800 cells taken randomly from two to three different eWAT sections per animal. The presence of macrophages in CLS was detected using anti-Mac2 antibodies. The abundance of dying adipocytes marked by CLS in eWAT sections was expressed as % of all adipocytes. In immunofluorescence analyses, perilipin 1-negative adipocytes, surrounded by macrophages (F4/80+ cells) that formed CLS, were considered to represent dying adipocytes. Within the CLS, proliferating (Ki67 stained) nuclei were identified and their relative (% of all 4′,6-diamidine-2′-phenylindole dihydrochloride (DAPI) positive nuclei) content was quantified. All analyses were performed using the imaging software NIS-Elements AR3.0 (Laboratory Imaging, Prague, Czech Republic). For the primary and secondary antibodies specification, see Supplementary Table S7. 

### 4.7. Statistical Analysis 

All values are presented as means ± SD. Comparisons were judged to be significant at *p* < 0.05. Data were analyzed by analysis of variance (one-way or two-way ANOVA). SigmaStat 3.5 software (Systat Software, San Jose, CA, USA) was used for statistical evaluation. Logarithmic, square-root or reciprocal transformations were used to stabilize variance or normality of samples when necessary. PCA, a multivariate statistical analysis, was used for the lipidomic data evaluation. Analysis was performed using the statistical software SIMCA-P+12 (Umetrics AB, Malmo, Sweden). 

## Figures and Tables

**Table 1:** Effects of eicosapentaenoic acid (EPA)/docosahexaenoic acid (DHA) on body weight, epididymal white adipose tissue (eWAT) weight, and DNA content.

	Week 1			Week 8		
	STD	HFD	HFF	STD	HFD	HFF
BW initial (g)	29.3 ± 1.9	29.6 ± 1.4	30.0 ± 2.4	28.1 ± 1.8 ^a^	28.1 ± 1.9 ^a^	28.2 ± 1.7 ^a^
BW dissection (g)	29.9 ± 1.9	33.9 ± 2.1 ^c^	33.9 ± 2.8 ^c^	34.0 ± 3.4 ^a^	48.8 ± 4.6 ^a,c^	47.0 ± 3.4 ^a,c^
BW gain (g)	0.7 ± 0.7	4.4 ± 0.8 ^c^	3.9 ± 1.1 ^c^	5.9 ± 2.9 ^a^	20.6 ± 3.8 ^a,c^	18.9 ± 3.1 ^a,c^
eWAT weight (mg)	509 ± 175	1084 ± 207 ^c^	1006 ± 284 ^c^	877 ± 365 ^a^	2310 ± 301 ^a,c^	1816 ± 254 ^a,b,c^
eWAT DNA (μg/depot)	194 ± 18	267 ± 47 ^c^	192 ± 30 ^b^	440 ± 98 ^a^	1313 ± 328 ^a,c^	1094 ± 183 ^a,b,c^
Adipocytes DNA (μg/depot)	n.d.	n.d.	n.d.	274 ± 67	541 ± 140^c^	445 ± 126

Mice were fed by standard (STD), high-fat diet (HFD), or a high-fat diet supplemented with omega-3 PUFA (HFF) and sacrificed at Week 1 or Week 8. Initial body weight (BW), BW at dissection, BW gain and weight of eWAT were evaluated. DNA was quantified in eWAT. Data are means ± SD; *n* = 8 for Week 1, *n* = 16–26 for Week 8. DNA content was also determined in collagenase-liberated adipocytes from eWAT at Week 8 (*n* = 8). ^a^ Significantly different from Week 1 between mice given same diet; ^b^ significantly different from the HFD group for the same period of dietary intervention; ^c^ significantly different from the STD group for the same period of dietary intervention; n.d., not determined.

## Supplementary Materials

The following are available online at http://www.mdpi.com/1660-3397/16/12/515/s1, Supplementary Table S1: Effects of the omega-3 PUFA supplementation on lipid mediators evaluated in eWAT extracts; Supplementary Table S2: Effect of the omega-3 PUFA supplementation on relative mRNA levels of the genes for enzymes involved in metabolism of polyunsaturated fatty acids; Supplementary Table S3: Antibodies used for flow cytometry; Supplementary Table S4: Specific panels of markers used to identify different cell populations; Supplementary Table S5: Effect of the omega-3 PUFA supplementation on relative mRNA levels of the genes for enzymes involved in adipogenesis; Supplementary Table S6: Sequences of primers; Supplementary Table S7: Antibodies used for immunohistochemistry; Supplementary Figure S1: Morphology and immunohistochemistry of eWAT at Week 1; Supplementary Figure S2 Percentage of proliferating leukocytes and macrophages in SVF of eWAT of mice fed HFD or HFF diet for 1 or 8 weeks determined using flow cytometry; Supplementary Figure S3 Percentage of proliferating CD45- cells, progenitors, preadipocytes and endothelial cells in SVF of eWAT of mice fed HFD or HFF diet for 1 or 8 weeks determined using flow cytometry; Supplementary Figure S4 Flow cytometry analysis of cells subsets in SVF isolated from eWAT.
