# Supplementary material for: Reduced Number of Adipose Lineage and Endothelial Cells in Epididymal fat in Response to Omega-3 PUFA in Mice Fed High-Fat Diet
Source: Mar Drugs. 2018 Dec 18;16(12):515. doi: 10.3390/md16120515 (PMC6316446; doi:10.3390/md16120515)
Supplement: Supplementary file 1 [file marinedrugs-16-00515-s001.pdf]

Supplementary Information

Supplementary Table S1 Effects of the omega-3 PUFA supplementation on lipid mediators evaluated in eWAT extracts.

		Week 1		Week 8	
		HFD	HFF	HFD	HFF
**AA-derived**	11,12-DiHETrE	1.00 ± 0.61	0.20 ± 0.11 [b]	2.58 ± 2.46	0.26 ± 0.22 [b]
	14,15-DiHETrE	1.00 ± 0.71	0.19 ± 0.07 [b]	1.77 ± 2.26	0.08 ± 0.03 [b]
	5-HETE	1.00 ± 0.41	0.84 ± 0.25	1.48 ± 0.28 [a]	0.45 ± 0.24 [a,b]
	8-HETE	1.00 ± 0.46	0.57 ± 0.08 [b]	2.22 ± 1.31 [a]	0.85 ± 0.39 [a,b]
	11-HETE	1.00 ± 0.52	0.56 ± 0.24 [b]	2.44 ± 1.27 [a]	0.93 ± 0.24 [a,b]
	12-HETE	1.00 ± 0.72	0.54 ± 0.32 [b]	2.09 ± 1.29	0.51 ± 0.15 [b]
	15-HETE	1.00 ± 0.69	0.48 ± 0.25 [b]	1.58 ± 0.75	0.70 ± 0.38 [b]
	PGD2	1.00 ± 0.49	0.70 ± 0.35 [b]	5.06 ± 3.16 [a]	2.69 ± 2.15 [a,b]
	PGE2	1.00 ± 0.62	0.55 ± 0.21[b]	1.53 ± 0.65 [a]	1.06 ± 0.52 [a,b]
	6-Keto-PGF1a	1.00 ± 0.59	0.79 ± 0.47	2.95 ± 1.13 [a]	1.35 ± 0.48 [b]
	11-Dh-TXB2	1.00 ± 0.24	1.08 ± 0.36	2.47 ± 0.63 [a]	1.68 ±0.23[a,b]
**ALA-derived**	9,10-DiHODE	1.00 ± 0.53	0.50 ± 0.23 [b]	0.12 ± 0.05 [a]	0.08 ± 0.05 [a,b]
	15,16-DiHODE	1.00 ± 0.42	0.61 ± 0.28 [b]	0.40 ± 0.18 [a]	0.26 ± 0.13 [a,b]
	9-HOTrE	1.00 ± 0.43	0.70 ± 0.28	0.88 ± 0.35	0.95 ± 0.75
	13-HOTrE	1.00 ± 0.55	0.67 ± 0.34 [b]	2.57 ± 1.66 [a]	1.22 ± 1.11 [a,b]
**DGLA-derived**	15-HETrE	1.00 ± 0.62	0.65 ± 0.35 [b]	2.37 ± 1.34 [a]	0.78 ± 0.24 [a,b]
**DHA-derived**	19,20-DiHDPA	1.00 ± 0.54	1.11 ± 0.49	1.28 ± 1.64	0.60 ± 0.21
	4-HDHA	1.00 ± 0.23	1.00 ± 0.34	2.57 ± 0.40 [a]	2.02 ± 0.66 [a]
	7-HDHA	1.00 ± 0.20	0.97 ± 0.32	2.18 ± 0.60 [a]	1.95 ± 0.56 [a]
	14-HDHA	1.00 ± 0.44	1.21 ± 0.85	1.01 ± 0.55	1.99 ± 1.38
	17-HDHA	1.00 ± 0.36	1.13 ± 0.74	1.14 ± 0.40	1.84 ± 1.07
**EPA-derived**	14,15-DiHETE	1.00 ± 0.70	2.87 ± 1.59 [b]	0.24 ± 0.23 [a]	0.35 ± 0.14 [a]
	17,18-DiHETE	1.00 ± 0.73	2.23 ± 1.27 [b]	0.16 ± 0.08 [a]	0.48 ± 0.15 [a,b]
	17,18-EpETE	1.00 ± 0.73	2.80 ± 1.30 [b]	0.73 ± 0.50	1.20 ± 0.93 [a]
	5-HEPE	1.00 ± 0.60	2.13 ± 1.27 [b]	0.32 ± 0.22	2.01 ± 0.73 [b]
	12-HEPE	1.00 ± 0.50	1.78 ± 1.24	0.68 ± 0.50	3.44 ± 2.63 [b]
	15-HEPE	1.00 ± 0.44	1.62 ± 1.16 [b]	1.14 ± 1.37	2.63 ± 2.15 [b]
	18-HEPE	1.00 ± 0.63	1.96 ± 1.18 [b]	0.74 ± 0.44 [a]	0.92 ± 0.46 [a,b]
**LA-derived**	9,10-DiHOME	1.00 ± 0.57	0.56 ± 0.27 [b]	0.26 ± 0.14 [a]	0.15 ± 0.06 [a,b]
	12,13-DiHOME	1.00 ± 0.56	0.57 ± 0.25 [b]	0.19 ± 0.08 [a]	0.13 ± 0.05 [a,b]
	9-HODE	1.00 ± 0.48	0.60 ± 0.18	1.81 ± 0.48 [a]	1.90 ± 1.47 [a]
	13-HODE	1.00 ± 0.59	0.52 ± 0.23 [b]	1.43 ± 0.86 [a]	1.00 ± 0.63 [a,b]
	13-Oxo-ODE	1.00 ± 0.57	0.51 ± 0.13 [b]	3.48 ± 1.98 [a]	2.49 ± 1.54 [a,b]

Abbreviations: AA, arachidonic acid; ALA, α-linolenic acid; DGLA, dihomo-γ-linolenic acid; DHA, docosahexaenoic acid; EPA, eicosapentaenoic acid; LA, linoleic acid; DiHETrE, dihydroxy-eicosatrienoic acid; HETE, hydroxy-eicosatetraenoic acid; HETrE, hydroxy-eicosatrienoic acid; PGD2, prostaglandin D2; PGE2, prostaglandin E2; PGF1a, prostaglandin 1α; Dh-TXB2, dehydro-thromboxane B2; DiHODE, dihydroxy-octadecadienoic acid; HOTrE, hydroxy-octadecatrienoic acid; DiHDPE, dihydroxy-docosapentaenoic acid; HDHA, hydroxy-docosahexaenoic acid; DiHETE, dihydroxy-eicosatetraenoic acid; EpETE, epoxy-eicosatetraenoic acid; HEPE, hydroxy-eicosapentaenoic acid; DiHOME, dihydroxy-octadecenoic acid; HODE, hydroxy-octadecadienoic acid; OxoODE, oxo-octadecadienoic acid. Data were expressed per whole eWAT depot and then normalized to the HFD mice at Week 1 mice. Data are means ± SD; n = 8–10. [a] Significant difference compared to Week 1 for mice with the same diets, [b] significant difference between the diets for the same period of the dietary intervention.

Supplementary Table S2 Effect of the omega-3 PUFA supplementation on relative mRNA. levels of the genes for enzymes involved in metabolism of polyunsaturated fatty acids.

A

	**Week 1**		**Week 8**	
	**HFD**	**HFF**	**HFD**	**HFF**
Alox5	1.00 ± 0.21	0.90 ± 0.20	0.59 ± 0.15 [a]	0.60 ± 0.22 [a]
Alox12	1.00 ± 0.22	1.13 ± 0.52	1.82 ± 0.88 [a]	2.55 ± 0.85 [a]
Alox15	1.00 ± 0.81	0.69 ± 0.34	0.30 ± 0.16 [a]	0.45 ± 0.27 [a]
15Pgdh	1.00 ± 0.06	1.02 ± 0.06	1.34 ± 0.08 [a]	1.58 ± 0.07 [a]

B

	**SVF**		**ADI**	
	**HFD**	**HFF**	**HFD**	**HFF**
Alox5	1.00 ± 0.78	0.72 ± 0.27	0.11 ± 0.03	0.10 ± 0.03
Alox12	1.00 ± 0.53	1.24 ± 0.46	0.16 ± 0.08	0.26 ± 0.20
Alox15	1.00 ± 0.48	1.00 ± 0.53	0.19 ± 0.06	0.18 ± 0.03
15Pgdh	1.00 ± 0.56	0.65 ± 0.15 [b]	1.07 ± 0.23	0.80 ± 0.20 [b]

Abbreviations: *15Pgdh*, gene for 15-hydroxyprostaglandin dehydrogenase; *Alox*, gene for lipoxygenase. (**A**) eWAT of the HFD or HFF mice at Week 1 or at Week 8. (**B**) SVF or adipocytes (ADI) isolated from eWAT of the HFD or HFF mice at Week 8. Data were normalized to the geometrical mean of two reference genes *Hprt*, *EF1a* for the whole eWAT mRNA, and *EF1a* and *Rn18s* for SVF and ADI mRNA. Data were expressed relative to those in eWAT of the HFD mice at Week 1 (**A**) or SVF of the HFD mice at Week 8 (**B**). Data are means ± SD; n = 8–10. [a] Significant difference compared to Week 1 for mice with the same diet, [b] significant difference between the diets for the same period of dietary intervention.

Supplementary Table S3 Antibodies used for flow cytometry.

Antigen	Fluorophore	Sources	Catalogue Number	Dilution
CD45	PerCP	BD Biosciences	552991	1:160
CD31	APC	BD Biosciences	553932	1:100
CD34	BV421	BD Biosciences	562608	1:200
Sca1	BV510	BioLegend	108129	1:300
CD24	PE	BD Biosciences	553262	1:600
CD11b	APC-Cy7	BD Biosciences	552773	1:300
F4/80	Biotin	eBioscience	13-4801-81	1:40
CD206	FITC	GeneTex	GTX43682	1:40
CD11c	PE	Affymetrix, eBioscience	12-0114	1:200
Ki67	BV605	BioLegend	652413	1:100
Streptavidin	eFluor710	Affymetrix, eBioscience	49-4317	1:160

Supplementary Table S4 Specific panels of markers used to identify different cell populations.

Cell type	Markers	References
Leukocytes	$CD45^{+}$	[1]
Macrophages	$CD45^{+}$ $F4/80^{+}$ $CD11b^{+}$	[2]
M1 macrophages	$CD45^{+}$ $F4/80^{+}$ $CD11b^{+}$ $CD11c^{+}$ $CD206^{-}$	[2]
M2 macrophages	$CD45^{+}$ $F4/80^{+}$ $CD11b^{+}$ $CD11c^{-}$ $CD206^{+}$	[2]
Double positive macrophages	$CD45^{+}$ $F4/80^{+}$ $CD11b^{+}$ $CD11c^{+}$ $CD206^{+}$	[2–4]
Double negative macrophages	$CD45^{+}$ $F4/80^{+}$ $CD11b^{+}$ $CD11c^{-}$ $CD206^{-}$	[2,4–6]
Endothelial cells	$CD45^{-}$ $CD31^{+}$	[7]
Progenitors	$CD45^{-}$ $CD31^{-}$ $CD34^{+}$ $Sca1^{+}$ $CD24^{+}$	[7,8]
Preadipocytes	$CD45^{-}$ $CD31^{-}$ $CD34^{+}$ $Sca1^{+}$ $CD24^{-}$	[7,8]

Supplementary Table S5 Effect of the omega-3 PUFA supplementation on relative mRNA. levels of the genes for enzymes involved in adipogenesis

A

	Week 1		Week 8	
	**HFD**	**HFF**	**HFD**	**HFF**
Pparg	1.00 ± 0.21	0.91 ± 0.22	0.60 ± 0.19 [a]	0.62 ± 0.11 [a]
Pdgfra	1.00 ± 0.21	1.20 ± 0.19	1.61 ± 0.65 [a]	1.84 ± 0.46 [a]
Pdgfrb	1.00 ± 0.30	1.03 ± 0.44	1.95 ± 0.59 [a]	1.60 ± 0.42 [a]
Sca1	1.00 ± 0.10	0.95 ± 0.15	0.97 ± 0.26	0.98 ± 0.38
Pref1	1.00 ± 0.46	0.98 ± 0.49	0.65 ± 0.35 [a]	0.30 ± 0.09 [a,b]
Cebpa	1.00 ± 0.17	1.04 ± 0.18	0.68 ± 0.16 [a]	0.80 ± 0.09 [a]

B

	SVF		ADI	
	**HFD**	**HFF**	**HFD**	**HFF**
Pparg	1.00 ±0.26	0.71 ± 0.14 [b]	2.97 ± 0.75	2.32 ± 0.69
Pdgfra	1.00 ±0.40	1.23 ± 0.19	0.66 ± 0.26	0.49 ± 0.07
Pdgfrb	1.00 ±0.29	0.72 ± 0.19 [b]	0.06 ± 0.04	0.08 ± 0.07
Sca1	1.00 ±0.48	0.90 ± 0.23	0.12 ± 0.06	0.17 ± 0.08
Pref1	1.00 ±0.61	0.41 ± 0.25 [b]	0.10 ± 0.09	0.15 ± 0.09
Cebpa	1.00 ±0.13	0.86 ± 0.20	4.46 ± 1.22	4.33 ± 1.04

Abbreviations: *Pparg*, peroxisome proliferator-activated receptor γ gene; *Pdgfra*, platelet-derived growth factor receptor α gene; *Pdgfrb*, platelet-derived growth factor receptor β gene; *Sca1*, stem cells antigen-1 gene; *Pref1*, preadipocyte factor 1 gene; *Cebpa*, CCAAT/enhancer-binding protein α gene. (**A**) eWAT of the HFD or HFF mice at Week 1 or at Week 8. (**B**) SVF or adipocytes (ADI) isolated from eWAT of the HFD or HFF mice at Week 8. Data were normalized to the geometrical mean of two reference genes *Hprt*, *EF1a* for whole eWAT mRNA, and *EF1a* and *Rn18s* for SVF and ADI mRNA. Data were expressed relative to those in eWAT of the HFD mice at Week 1 (**A**) or SVF of the HFD mice at Week 8 (**B**). Data are means ± SD; n = 8-10. [a]Significant difference compared to Week 1 for mice with the same diet, [b]significant difference between the diets for the same period of dietary intervention.

Supplementary Table S6 Sequences of primers.

Gene Name	Gene ID	5´Sequence	3´Sequence
5Lox	11689	TCAAACGATCACCCACCTTCTGC	TTCCCGGGCCTTAGTGTTGATAGC
12Lox	11685	GCAGGCCTGGTGTCGGGAGAT	GCTGGGCAGTGCATGTGAAAAC
15Lox	11687	ATCAGGGGGACACAATGAGC	CTTCCGTGCACCCTGTTTTT
15Pgdh	15446	TCCGGGACCTGCAAGCGAA	TGTCACTGGGACACAGCCACAC
18SRna	19791	GCCCGAGCCGCCTGGATAC	CCGGCGGGTCATGGGAATAAC
Arg1	11846	TCCAACTGCCAGACTGTGGTC	ACGGGGACCTGGCCTTTGTT
CCl2	20296	CATGCTTCTGGGCCTGCTGTT	CCTGCTGCTGGTGATCCTCTTGTA
CCr2	12772	GTGGTTTGTGGTCTGTGGGCTT	GGAGTTCCCACTCACTCAAAGGACA
Cebpa	12606	ACGGGGACCATTAGCCTTGTGT	CTCCTTCCCCCAGTCGTTAGTG
EF1a	13627	TGACAGCAAAAACGACCCACCAAT	GGGCCATCTTCCAGCTTCTTACCA
Hprt	15452	GCTGAGGCGGCGAGGGAGAG	GCTAATCACGACGCTGGGACTGC
IFNg	15978	GCCAAGTTTGAGGTCAACAACCCA	CCACCCCGAATCAGCAGCGA
IL1b	16176	TCCCCCACACGTTGACAGCTAGG	TCGGCCAAGACAGGTCGCTCA
Ki67	17345	ACTGGAGGTGAAAACCACACT	AGGGTAACTCGTGGAACCAA
Nos2	18126	CTTTGCCACGGACGAGACGGATAGG	CGGGCACATGCAAGGAAGGGAACT
Pcna	18538	ACTCTACAACAAGGGGCACA	AGTGGAGAGCTTGGCAATGG
Pdgfra	18595	CAGGAAGGCGTAGGGAATCAGGT	TCTCACCTCACATCTGGTCGGC
Pdgfrb	18596	CCCCCAGCCTTGCCAGTTCCA	TGGGCAGCCTGAATCCTGTGG
Pparg	19016	GCCTTGCTGTGGGGATGTCTC	CTCGCCTTGGCTTTGGTCAG
Pref1	13386	CCCCTGGCTGTGTCAATGGAGTCT	ATTGTTGGCGCAGGGGGTTGAGGT
Sca1	110454	GGAACATTGCAGGACCCCAGA	ATGACCCTGGAGGCACACAGC
Tgfb	21803	GTGGCTGAACCAAGGAGACGGAA	CTCTCCGGTGCCGTGAGCTG
Tnfa	21926	AGCTGTCCCCACCTGGCCTCTC	CCCGTGGGGAGCAGAGGTTCAGT

Supplementary Table S7 Antibodies used for immunohistochemistry.

Primary antibody	Company	Catalog number	Secondary antibody	Company	Catalog number
anti-Mac2	Cedarlane	CL8942AP	anti-mouse IgG biotinylated secondary antibody	Vector Labs	BA-2000
anti-F4/80	Santa Cruz	sc-377009	anti-mouse IgG, Alexa Fluor 488 conjugate	Invitrogen	A10680
anti-Ki-67	Abcam	ab15580	anti-rabbit IgG, Alexa Fluor 555 conjugate	Invitrogen	A21428
anti-Perilipin 1	Abcam	ab61682	anti-goat IgG, Alexa Fluor 633 conjugate	Invitrogen	A21082

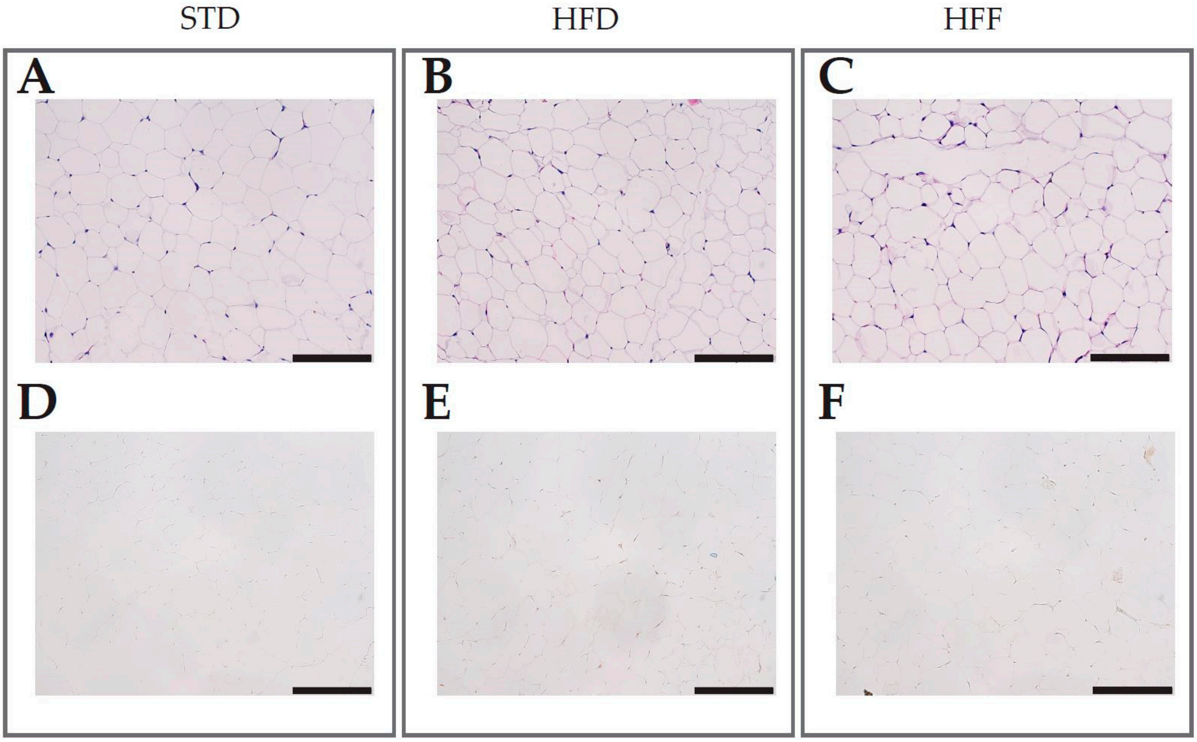

Supplementary Figure 1 Morphology and immunohistochemistry of eWAT at Week 1. Representative histological sections of eWAT from mice fed STD (**A**, **D**), HFD (**B, E**) or HFF (**C, F**). Hematoxylin and eosin staining for morphometry of adipocytes (**A, B, C**), as evaluated in Fig. **1D** in the main text. Immunohistochemical staining using macrophage marker MAC2 for quantification of CLS (**D, E, F**), as evaluated in Fig. 1**H** in the main text. n = 6-8. Bar represents 200 μm.

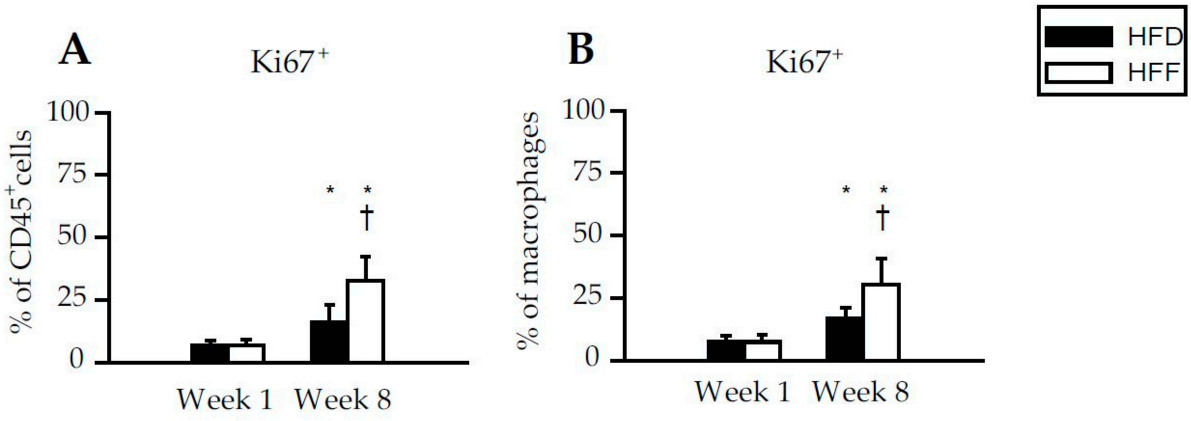

Supplementary Figure S2 Percentage of proliferating leukocytes and macrophages in SVF of eWAT of mice fed HFD or HFF diet for 1 or 8 weeks determined using flow cytometry. Graphs are repploted data from Fig. 4A and 4B in the main text. Data are means ± SD; n = 6–8. * Significant difference between mice with the same diet; † significant difference from HFD at Week 8.

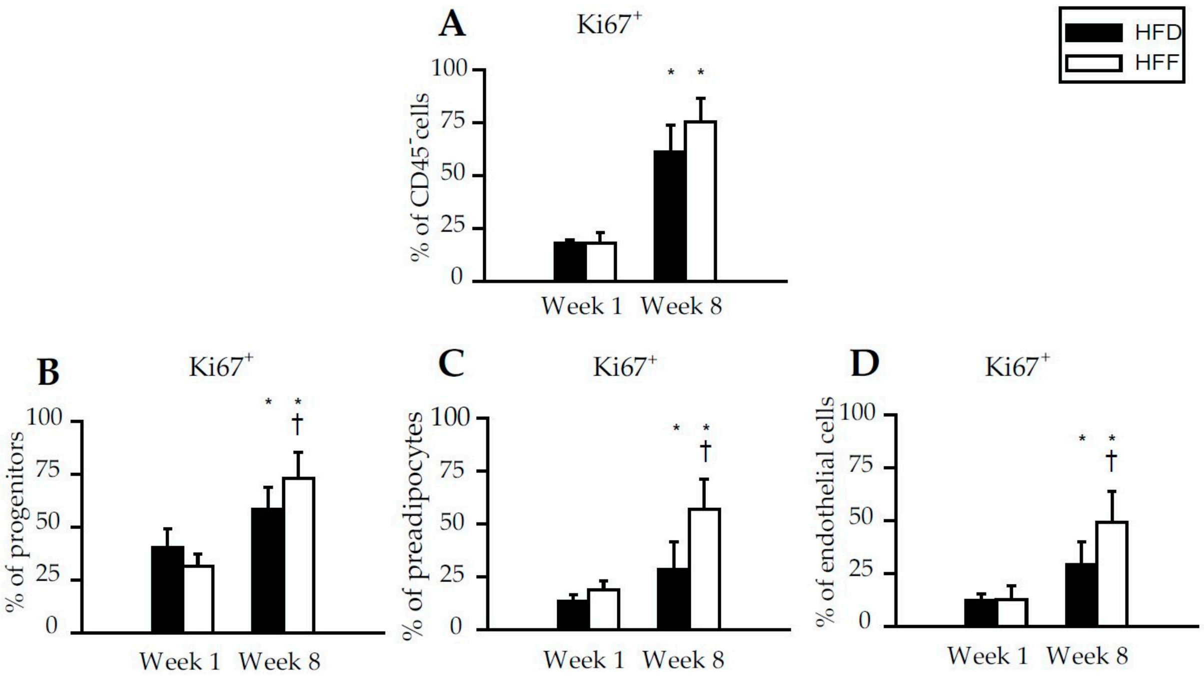

Supplementary Figure S3 Percentage of proliferating $CD45^{-}$ cells, progenitors, preadipocytes and endothelial cells in SVF of eWAT of mice fed HFD or HFF diet for 1 or 8 weeks determined using flow cytometry. Graphs are repploted data from Fig. 6A, 6B, 6C and 6D. Data are means ± SD; n = 6–8. * Significant difference between mice with the same diet; † significant difference from HFD at Week 8.

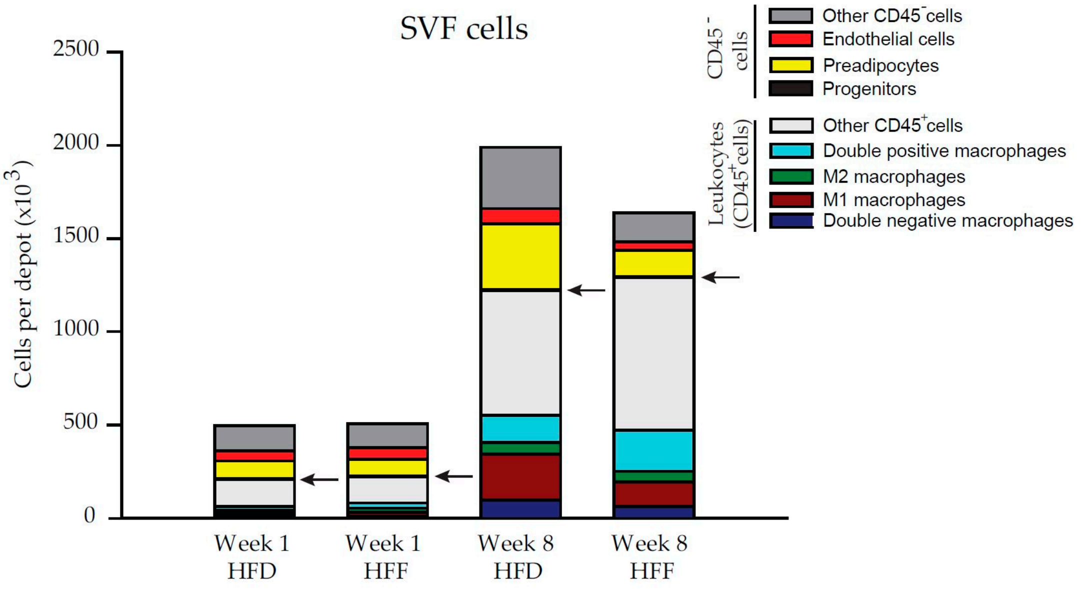

Supplementary Figure S4 Flow cytometry analysis of cells subsets in SVF isolated from eWAT. The numbers of cells are calculated per depot and re-plotted from Figures 4 and 6 data. Progenitors are indicated by arrows.

References

1. Baer, P.C. Adipose-derived mesenchymal stromal/stem cells: An update on their phenotype in vivo and in vitro. *World J Stem Cells* **2014**, *6*, 256–265, doi:10.4252/wjsc.v6.i3.256.
2. Morris, D.L.; Singer, K.; Lumeng, C.N. Adipose tissue macrophages: phenotypic plasticity and diversity in lean and obese states. *Curr Opin Clin Nutr Metab Care* **2011**, *14*, 341–346, doi:10.1097/MCO.0b013e328347970b.
3. Wentworth, J.M.; Naselli, G.; Brown, W.A.; Doyle, L.; Phipson, B.; Smyth, G.K.; Wabitsch, M.; O'Brien, P.E.; Harrison, L.C. Pro-inflammatory CD11c+CD206+ adipose tissue macrophages are associated with insulin resistance in human obesity. *Diabetes* **2010**, *59*, 1648–1656, doi:10.2337/db09-0287.
4. Garg, S.K.; Delaney, C.; Shi, H.; Yung, R. Changes in adipose tissue macrophages and T cells during aging. *Crit Rev Immunol* **2014**, *34*, 1–14.
5. Zeyda, M.; Gollinger, K.; Kriehuber, E.; Kiefer, F.W.; Neuhofer, A.; Stulnig, T.M. Newly identified adipose tissue macrophage populations in obesity with distinct chemokine and chemokine receptor expression. *Int J Obes (Lond)* **2010**, *34*, 1684–1694, doi:10.1038/ijo.2010.103.
6. Lumeng, C.N.; Liu, J.; Geletka, L.; Delaney, C.; Delproposto, J.; Desai, A.; Oatmen, K.; Martinez-Santibanez, G.; Julius, A.; Garg, S., et al. Aging is associated with an increase in T cells and inflammatory macrophages in visceral adipose tissue. *Journal of immunology* **2011**, *187*, 6208–6216, doi:10.4049/jimmunol.1102188.
7. Berry, R.; Rodeheffer, M.S. Characterization of the adipocyte cellular lineage in vivo. *Nature cell biology* **2013**, *15*, 302–308, doi:10.1038/ncb2696.
8. Rodeheffer, M.S.; Birsoy, K.; Friedman, J.M. Identification of white adipocyte progenitor cells in vivo. *Cell* **2008**, *135*, 240–249, doi:10.1016/j.cell.2008.09.036.
